# Insulin Resistance and Cancer: In Search for a Causal Link

**DOI:** 10.3390/ijms222011137

**Published:** 2021-10-15

**Authors:** Eusebio Chiefari, Maria Mirabelli, Sandro La Vignera, Sinan Tanyolaç, Daniela Patrizia Foti, Antonio Aversa, Antonio Brunetti

**Affiliations:** 1 Department of Health Sciences, University of “Magna Græcia” Catanzaro, 88100 Catanzaro, Italy; echiefari@gmail.com (E.C.); maria.mirabelli@unicz.it (M.M.); 2 Department of Clinical and Experimental Medicine, University of Catania, 95100 Catania, Italy; sandrolavignera@unict.it; 3 Department of Internal Medicine, Division of Endocrinology and Metabolism, School of Medicine, Biruni University, Istanbul 34010, Turkey; stanyolac@gmail.com; 4 Department of Experimental and Clinical Medicine, University of “Magna Græcia” Catanzaro, 88100 Catanzaro, Italy; foti@unicz.it (D.P.F.); aversa@unicz.it (A.A.)

**Keywords:** insulin resistance, hyperglycemia, cancer, epigenetics, gut microbiota, metformin, PPARγ

## Abstract

Insulin resistance (IR) is a condition which refers to individuals whose cells and tissues become insensitive to the peptide hormone, insulin. Over the recent years, a wealth of data has made it clear that a synergistic relationship exists between IR, type 2 diabetes mellitus, and cancer. Although the underlying mechanism(s) for this association remain unclear, it is well established that hyperinsulinemia, a hallmark of IR, may play a role in tumorigenesis. On the other hand, IR is strongly associated with visceral adiposity dysfunction and systemic inflammation, two conditions which favor the establishment of a pro-tumorigenic environment. Similarly, epigenetic modifications, such as DNA methylation, histone modifications, and non-coding RNA, in IR states, have been often associated with tumorigenesis in numerous types of human cancer. In addition to these observations, it is also broadly accepted that gut microbiota may play an intriguing role in the development of IR-related diseases, including type 2 diabetes and cancer, whereas potential chemopreventive properties have been attributed to some of the most commonly used antidiabetic medications. Herein we provide a concise overview of the most recent literature in this field and discuss how different but interrelated molecular pathways may impact on tumor development.

## 1. Introduction

The reduced response of peripheral target tissues to insulin action leads to insulin resistance (IR), a condition that is characterized by a compensatory increase of circulating insulin levels to maintain euglycemia. When the compensatory increase of insulin production can no longer compensate for IR, blood sugar rises and the insidious process leading to type 2 diabetes mellitus begins [1,2]. In this context, the liver, the major site of insulin clearance, plays a crucial role in ensuring euglycemia and insulin sensitivity [3,4]. Apart from type 2 diabetes, IR is also a key component of metabolic syndrome, a cluster of individual disorders all predisposing to cardiovascular disease [5,6]. Over the last decades, the relationship between IR-related diseases and cancer has generated wide-ranging interest, and extensive research has been conducted to elucidate the underlying mechanisms for this link. However, although the pivotal role of IR in cancer is well acknowledged, and many plausible explanations have been provided, the models proposed so far to explain these phenomena do not exhaustively explain the causal association between IR and cancer. The issue is complicated further by the fact that the association seen in epidemiological studies between IR-related conditions and cancer may depend on several mechanisms, and not necessarily the same mechanisms for all types of cancer which may affect IR. For instance, overweight/obese men with IR appear to be at higher risk of getting prostate cancer, whereas an inverse correlation has been observed in patients with type 2 diabetes, thus suggesting that different mechanisms might link IR to prostate cancer development in these individuals [7]. In addition, a divergence in time trends in the incidence of cancer and other IR-related diseases has been observed: in contrast to that of obesity and type 2 diabetes, the incidence rate of cancer (all sites), which had been steadily increasing since the early 1970s, has currently plateaued [8]. In the present review, we summarize the current status of knowledge on the connections between IR, obesity, type 2 diabetes, and cancer development, as well as the contribution of a series of molecules and pathways in both metabolic dysfunction and cancer risk.

## 2. Insulin Receptor Signaling and Cancer

**Figure 1:**
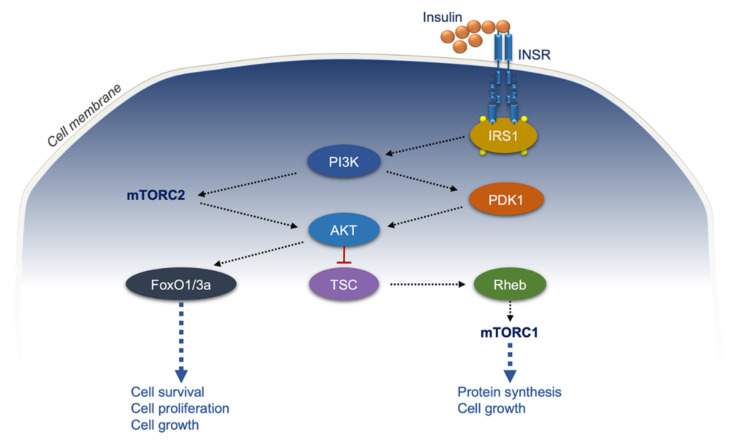
Insulin receptor signaling and cancer: scheme. INSR, insulin receptor; IRS1, INSR substrate 1; PI3K, phosphoinositide 3-kinase; PDK1, phosphoinositide-dependent protein kinase-1; AKT, Ak strain transforming/protein kinase B; FoxO1/3a, forkhead box protein O1/3a; TSC, tuberous sclerosis complex; Rheb, Ras homolog enriched in brain; mTORC1/2, mammalian target of rapamycin complexes; black dotted arrows, activation; blue dotted arrows, biological effects of the downstream signaling.

IR is determined by a complex interplay of genetic and environmental triggers. So far, a variety of genetic alterations leading to defects in insulin receptor (INSR) and/or other components of the insulin signaling pathway have been recognized as significant causes of uncommon forms of IR and diabetes [9]. Instead, abnormal visceral fat accumulation from reduced physical activity and excess caloric intake in genetically susceptible people is considered the main driver of IR in humans worldwide. As such, the majority of obese subjects are resistant to insulin action, and most of them experience a recovery of insulin sensitivity after weight loss, independently from the therapeutic strategy used [10,11,12,13,14,15]. In addition, the reduction of INSR has been observed in obese rodents and humans, in both adipocytes and other cell types [16,17,18,19,20]. There are two INSR isoforms deriving from an alternative splicing process: the isoform A (INSR-A) and the isoform B (INSR-B). These two alternatively spliced INSR isoforms differ in the presence or absence of exon 11, which encodes a segment of 12 amino acids in the C-terminal end of the α subunit in INSR-B [21,22,23]. The absence of exon 11 in the INSR-A isoform allows for distinct functional properties, such as the recognition and binding, not only of insulin, but also of pro-insulin and insulin-like growth factors (IGFs), with a greater affinity for IGF2 than IGF1 [24,25]. The INSR-B isoform is an insulin-specific receptor that mediates glucose uptake in insulin target tissues and is mainly involved in metabolic processes [26]. However, INSR-A has 1.7-fold higher affinity for insulin and is internalized and recycled faster than INSR-B [27,28,29]. INSR-A is the predominant isoform expressed in embryonic and fetal tissues, as it regulates intrauterine growth. Furthermore, given its involvement in mitogenic signaling pathways, INSR-A is overexpressed in various malignant cells [30,31,32,33]. To better understand the molecular mechanisms that regulate *INSR* gene expression, the human *INSR* gene promoter has been identified and analyzed in depth by several groups [22,34,35]. Previously, we have demonstrated that the architectural transcription factor HMGA (high mobility group A1), known as a driver of neoplastic transformation, enhances the expression of the *INSR* gene and other genes involved in glucose metabolism [36,37]. By itself, HMGA1 is unable to activate gene transcription but, via binding to AT-rich motifs within gene promoters, can assemble DNA-protein complexes which are able to induce gene activation [35,38,39,40,41,42]. Qualitative and/or quantitative defects in HMGA1 protein or abnormalities in protein binding to consensus sequences within the *INSR* gene may affect *INSR* gene transcription [38,43,44,45,46]. On the other hand, upregulation of HMGA1, by inhibiting the transcriptional activity of the tumor suppressor p53, can induce uncontrolled activation of the *INSR* gene in cells that normally express low INSR levels, thereby amplifying the biological effects of insulin, thus triggering neoplastic transformation [47]. In this regard, the expression of INSR in human tumors has been reported, together with the observation that insulin can induce the growth of cancerous cells at both physiological and pharmacological concentrations [48,49,50]. Consistent with this, chronic sustained hyperinsulinemia can amplify the growth-promoting effects of insulin in patients with severe monogenic IR syndromes, most often due to mutation of the *INSR* gene, in which acanthosis nigricans, organomegaly, pseudo-acromegaloid soft tissue overgrowth, ovarian tumors, and colonic polyposis are the direct consequence of excessive circulating insulin [51,52,53]. Various mechanistic studies indicate that INSR exerts its oncogenic potential in malignant cells by activating multiple intracellular signaling pathways [54]. Recently, it has also been reported that several RNA-binding proteins involved in alternative splicing, such as CUGBP1, hnRNPH, hnRNPA1, hnRNPA2B1, and SF2/ASF, were upregulated in certain tumors in which the INSR-A:INSR-B ratio was found to be increased [55]. Both INSR isoforms can form heterodimers with the IGF1-R, generating INSR-A/IGF-1R and INSR-B/IGF-1R hybrid receptors [56]. Ligand binding to homodimeric INSR and IGF-1R, as well as to hybrid receptors, drives the activation of genes involved in cell growth, differentiation, survival, and proliferation (Figure 1).

In brief, insulin binding to either homodimeric INSR or hybrid receptors triggers the autophosphorylation of specific residues in the cytoplasmic tail of the receptor β subunit, and this is followed by the activation of effector proteins, such as INSR substrate (IRS) proteins, via Src homology 2 (SH2) and phosphotyrosine-binding (PTB) domains [57,58]. Then, the insulin signaling is amplified by the recruitment of phosphoinositide 3-Kinase (PI3K) to the plasma membrane and its activation into phosphatidylinositol (3,4,5)-trisphosphate (PIP3), followed by the phosphorylation of Akt/PKB at Thr308 residue via phosphoinositide-dependent protein kinase-1 (PDK1) [59,60,61]. In parallel, insulin activates the mammalian Target of Rapamycin Complexes 2 (mTORC2), which phosphorylates Akt/PKB at Ser 473, a critical step for the optimal activation of its kinase activity [62].

Once activated, Akt/PKB phosphorylates the tuberous sclerosis complex (TSC), blocking its GTPase activating protein activity toward the small GTPase Rheb, allowing the downstream stimulation of mTORC1.

Sustained mTORC1 activity, which may be caused by nutrients overload, promotes IR [63]. The PI3K/Akt/mTOR axis constitutes a crucial pathway regulating a plethora of biological processes involved in tumor development, such as angiogenesis, proliferation, metabolism, survival, and differentiation [64,65,66,67]. Thus, it is not surprising that the PI3K/Akt/mTOR pathway may represent a novel potential therapeutic target in cancer, in addition to being a prognostic and diagnostic parameter [68]. Akt/PKB also phosphorylates the Forkhead box protein O1 (FoxO1), a transcription factor involved in the regulation of cell proliferation and apoptosis. By inhibiting the glycogen synthase kinase 3 (GSK3), FoxO1 reinforces the action of proliferative signals through cyclin D1 [69].

**Figure 2:**
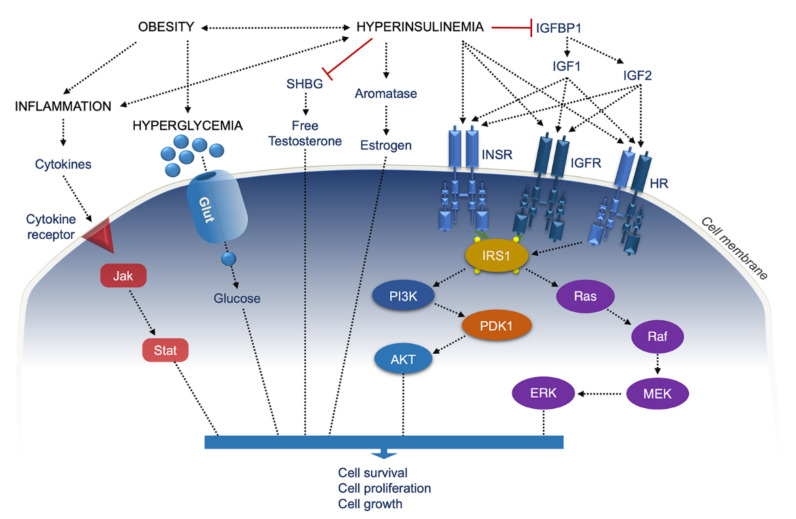
Multidimensional model through which obesity, hyperinsulinemia, and related inflammation influence tumor development. IGF1/2, insulin-like growth factor 1/2; IGFBP1, IGF binding protein 1, INSR, insulin receptor; IGFR, IGF receptor; HR, hybrid receptor; IRS1, INSR substrate 1; PI3K, phosphoinositide 3-Kinase; PDK1, phosphoinositide-dependent protein kinase-1; AKT, Ak strain transforming/protein kinase B; Ras, rat sarcoma; Raf, rapidly accelerated fibrosarcoma; MEK mitogen-activated protein kinase; ERK, extracellular signal-regulated protein kinase; Jak, janus kinase; STAT, signal transducer and activator of transcription; SHBG, sex-hormone binding globulin; red lines, inhibition.

Other mitogenic actions of insulin are mediated by the rat sarcoma-mitogen-activated protein kinase/ERK (Ras-MAPK/ERK) pathway, whose activation can lead to a broad range of cellular functions, including cell growth and proliferation, differentiation, and survival [70,71]. In addition to this, tumorigenesis can be mediated by other endocrine and/or metabolic mechanisms that are secondary to chronic sustained hyperinsulinemia. Among them, the IGF system plays a critical role. Actually, the effects of both IGF-1 and IGF-2 on cell proliferation, differentiation, and protection from apoptosis are well known. Under conditions of IR, hyperinsulinemia leads to decreased hepatic production of IGF-binding proteins 1 and 2 (IGFBP1/2) and increased tissue bioavailability of free IGF-1 and IGF-2, thus enhancing the activation of intracellular mitogenic signaling pathways at the expense of essential metabolic pathways in pre-malignant and malignant cells [72,73,74]. Several clinical studies in this context have found an association between IGF-1 and IGF-1:IGFBP3 ratio with the development of cancer [75]. In particular, the IGF-1–IGF-1R axis has been involved in the progression of breast, colorectal, prostate, and pancreatic cancer [76,77]. Besides the IGFBPs, hyperinsulinemia may also affect the hepatic production and secretion of the sex-hormones-binding globulin (SHBG), thereby increasing the bioavailability of sex steroids (estradiol and testosterone), which, in turn, positively influences tumor development and progression [75] (Figure 2).

In this regard, markedly higher circulating levels of the biologically active estrogen estradiol have been documented in women with polycystic ovary syndrome (PCOS), a hormonal disorder closely related to IR and hyperinsulinemia, in whom an increased risk of endometrial cancer was observed [50]. A synergistic effect of insulin and estrogen signaling is also involved in breast carcinogenesis, in which estrogens enhance insulin signaling by increasing both the expression and activity of molecules involved in the insulin/IGF-I pathway [78,79].

## 3. Diabetes and Cancer

The term “diabetes mellitus”, commonly known as “diabetes”, defines a group of metabolic disorders resulting from deficiencies in insulin secretion, action, or both [80]. In addition to its adverse effects on microvascular outcomes and the risk for cardiovascular mortality, diabetes is also a relevant risk factor for many cancers. Presently, the World Health Organization (WHO) estimates that about 422 million people worldwide have diabetes, and 1.6 million deaths can be directly attributed to diabetes each year [81]. An increasing amount of epidemiological evidence suggests that the incidence rate for cancer is increased in both type 1 and type 2 diabetes, and cancer is now regarded as a leading cause of death in patients with diabetes [82]. Table 1 reports the main results from selected meta-analyses, demonstrating the association between both type 1 and type 2 diabetes and increased risk of neoplastic disease [7,83,84,85,86,87,88,89,90,91,92,93,94,95,96,97,98,99,100,101,102,103,104,105,106,107,108]. The association between diabetes and increased incidence of cancer remained significant in some studies, thus consolidating the notion that pre-existing diabetes would worsen the prognosis for survival in cancer patients. Given the higher prevalence of type 2 diabetes and its age-related onset, the association of this form of diabetes with cancer is stronger compared to that of type 1 diabetes [109], although in this latter disease a significantly higher incidence of liver, pancreas, and kidney cancer, and endometrial and ovarian cancer has been found [110].

As stated above, IR is a key feature of type 2 diabetes [1], and virtually all individuals destined to become diabetic show a well-known condition of IR, which is initially overcome by increased beta-cell insulin secretion and plasma insulin concentrations [1,2]. Therefore, as emphasized in previous studies, we can hypothesize that among the potential pathogenetic mechanisms linking type 2 diabetes to cancer, chronic sustained hyperinsulinemia may be etiologically related to the development of cancer in affected patients. At present, the occurrence of hepatocellular neoplasms after intraportal pancreatic islet transplantation into diabetic rats is the only direct experimental evidence of a causative role for elevated insulin in the initiation phase of tumorigenesis [111]. Nevertheless, even if the relevance of insulin in tumor initiation is still a controversial issue, many in vivo and in vitro observations actually link hyperinsulinemia to cancer promotion and progression [112,113]. In addition, several cohort studies showed that subjects with high insulin levels have an increased risk for certain types of cancers, in addition to a poor prognosis [114,115,116,117,118,119,120]. A cancer-promoting effect of insulin has been observed even for exogenous insulin in patients with a long history of type 2 diabetes [110,121,122]. However, given that the autoimmune, insulinopenic form of type 1 diabetes may also be associated with a higher risk of certain types of cancer, it is possible that additional mechanisms can also contribute to the increased cancer risk in diabetes. Hyperglycemia is the common characterized metabolic alteration in both type 1 and type 2 diabetes. Although recent studies, in this context, have shown that not all cancer cell types rely on glucose for proliferation [123], many malignant tumors avidly take up glucose for cell survival and growth [124,125]. These data are consistent with observations from the milestone Me-Can cohort study estimating that for every 1 mmol/L increment in plasma glucose, the risk of incident cancer increased both in women (RR 1.11, 95% CI 1.05, 1.16) and in men (RR 1.05, 95% CI 1.01, 1.1), as well as the risk for fatal cancer (women: RR 1.21, 95% CI 1.11, 1.33; men: RR 1.15, 95% CI 1.07, 1.22) [126]. However, it remains unclear whether the effect of glucose on cancer growth would be independent from hyperinsulinemia, dyslipidemia, and the increased pro-inflammatory cytokines milieu commonly observed in patients with IR and type 2 diabetes [127]. Recently, a potential causality between genetically driven IR and greater risk of breast cancer has been established in a Mendelian randomization study in obese and physically inactive postmenopausal women, whereas genetically elevated fasting glucose levels were associated with a reduced risk [128]. Conversely, genetically increased fasting insulin levels, but not type 2 diabetes or dyslipidemia, were causally associated with increased risk of pancreas cancer [129].

## 4. Adipose Tissue, Obesity, Inflammation, and Cancer

The WHO estimates that in 2016, 1.9 billion adults aged 18 years and older were overweight. Of these, over 650 million were obese. In addition, over 340 million children and adolescents aged 5–19 were overweight or obese in 2016 [130]. According to research from the American Cancer Society, excess body weight is thought to be responsible for ~8% of all cancers in the United States, and ~7% of all cancer deaths [131]. The excess body fat that defines obesity is an independent risk factor for cancer incidence, cancer recurrence, and cancer-specific mortality among individuals diagnosed with several types of early-stage tumors [132,133,134]. The relationship between obesity and risk of cancer is complex and independent of gender and tumor site. Based on reports from the World Cancer Research Fund/American Institute for Cancer Research, there is strong evidence for a causal association between excess adiposity and cancer risk in six anatomic sites, comprising breast, esophagus (adenocarcinoma), pancreas, colon-rectum, endometrium, and kidney, whereas less evidence exists on the link between obesity and other tumors [135]. The role of obesity in colorectal carcinogenesis was initially documented in a large prospective multi-racial population of the U.S., where, after controlling for potential confounders, a positive linear association with risk of colorectal cancer death was found across the entire body mass index (BMI) spectrum. The increased risk resulting from a higher BMI category was more pronounced in men than in women [136]. Other studies confirmed the strong association between BMI and colorectal cancer in different countries [137,138,139]. However, subsequent evidence for a gendered connection was rather controversial, given that overweight and obese women enrolled in the large, prospective Nurses’ Health Study II were found to retain a 37% and 97%, respectively, increased risk of early-onset (prior to 50 years of age) colorectal cancer when compared to women from the normal reference BMI category [140]. The role of obesity in postmenopausal breast cancer has also been underscored and extensively studied, mostly focusing on adipose-tissue-derived adipokines and their specific functions. In postmenopausal women, obesity has been consistently associated with hormone receptor (HR)-positive breast tumor specific incidence and mortality, but not with the aggressive HR-negative or triple-negative subtypes [141,142]. Besides its energy-storage properties, adipose tissue represents a dynamic metabolically active secretory organ producing numerous functional adipokines, which regulate insulin sensitivity, appetite, inflammation, immunity, hematopoiesis, and angiogenesis in either physiological or aberrant manners. At a molecular level, in obesity, the shift of the adipocyte secretome to a pro-inflammatory profile is triggered by the hypoxic status of the adipose tissue of the obese via the hypoxia-inducible factor-1 (HIF-1) [143,144], a nuclear protein which, by interacting with HMGA1 and the transcription factor NF-kB, a master regulator of inflammation, may modulate the transcription of several relevant genes [145,146,147]. Indeed, in obese individuals, the enlarged visceral fat tissue possesses the ability to generate systemic pro-inflammatory and pro-oncogenic factors that increase cell susceptibility to cancer initiation or progression in many organs, including the breast [148]. Additionally, emerging evidence suggests that, among women, the breast adipose tissue itself participates in the crosstalk with breast cancer cells and contributes to tumorigenesis. Cancer-associated adipocytes, structurally characterized by small size and dispersed lipid droplets [149], are located in the invasive front of the breast tumor and evolve to accommodate tumor growth, participating in its dissemination through abnormal cell–cell interactions, generation of lipid-derived molecules and potentially mutagenic metabolites, and release of endocrine factors [150]. In human physiology, breast adipocytes are known for their high plasticity, as they undergo massive phenotypic and structural modifications during pregnancy and lactation, including reprogramming and de-differentiation into small pre-adipocytes, in order to fulfill the new maternal metabolic demands and support the growth and function of the adjacent epithelial ducts, which are the milk-producing structures [151]. Further, these cells communicate with stromal and immune components of the breast, and this may contribute to tumor development, as well as to the physiological pregnancy-lactation cycles and later involution of the gland. Some of the endocrine factors released by the mammary adipose cells may be also crucial for the function of the entire organism [150], as for local cancer cell proliferation, motility, invasiveness, epithelial to mesenchymal transition (EMT), stemness maintenance, tumor angiogenesis, and resistance to chemotherapy via activation of different molecular mechanisms [149,150,152]. Intriguingly, as reviewed in detail by Rybinska and colleagues, cancer-associated adipocytes share some common features with obese visceral abdominal adipocytes, such as the secretion of high levels of motility and extracellular matrix remodeling factors (e.g., CCL2, CCL5, autotaxin, MMPs), pro-inflammatory cytokines (e.g., IL-1β, IL-6, TNF-α, VEGF), and IR-associated adipokines (e.g., leptin, resistin) [153].

While circulating levels of classic pro-inflammatory adipokines and cytokines, such as leptin, resistin, TNF-α, and IL-6, are regularly increased in breast cancer patients, the insulin-sensitizing adiponectin, which exerts anti-proliferative, anti-migratory, and anti-apoptotic effects on tumor cells independent of their HR status, is considered protective against breast carcinogenesis, especially in postmenopausal women [154].

Leptin, the prototype of adipokines discovered in the mid-nineties, was initially proposed to mediate appetite and energy balance, with central anorexigenic actions. Circulating levels of leptin are directly proportional to the amount of body fat and particularly sensitive to caloric deprivation [155]. Women tend to have higher leptin levels than men, in view of different body fat distribution and sex hormone patterns, but a significant decline in the amount of circulating leptin is commonly observed after menopause [156]. Leptin binds to leptin receptors (Ob-Rs) located throughout the central nervous system, as well as in a variety of peripheral tissues and malignancies, including breast, endometrial, and gastrointestinal cancers [157,158,159]. Upon binding to Ob-Rs, leptin promotes cancer cells survival, proliferation, and metastasis, activating several signaling pathways, including MAPK/ERK, PI3K/Akt, and Janus kinase (Jak)-STAT signaling [89]. Leptin may further contribute to tumor development and metastasis by promoting the acceleration of EMT in cancer stem cells [160]. Remarkably, local leptin production is a better predictor of breast carcinogenesis and metastasis than circulating leptin levels [158]. In this context, it should be noted that generation and secretion of pro-inflammatory cytokines and adipokines, such as leptin and resistin, is higher in breast-cancer-associated adipocytes than in mature mammary adipocytes [161].

Resistin, originally referred as the adipose-tissue-specific secretory factor (ADSF), is an important adipokine that links obesity, inflammation, IR, and diabetes. Resistin promotes the ubiquitination and subsequent degradation of IRS1 and IRS2, and activates the downstream Jak-STAT pathway [162]. Recently, resistin has also been proposed as an early breast cancer biomarker. In this regard, studies from several research groups have indicated that resistin can support cancer growth and metastasis through Stat3 activation and by triggering the ezrin/radixin/moesin (ERM) protein family, which plays an essential role in cell migration and invasion [163,164,165,166]. Furthermore, similar to leptin, resistin promotes the metastatic potential of breast cancer cells by inducing EMT and stemness [167].

Adiponectin is the principal anti-inflammatory molecule produced by the adipose tissue, and to a lesser extent by skeletal muscle, heart, liver, bone marrow, and the central nervous system [154]. An antitumor action of adiponectin has been reported in T47D, MDA-MB-231, and MCF-7 adenocarcinoma cells, which are among the most reliable in vitro models of breast cancer [167]. It has been reported that adiponectin counteracts the leptin-induced migration and invasion of breast cancer cells [168,169]. At the molecular level, upon binding to its receptor in target tissues, adiponectin inhibits leptin-induced oncogenic signaling through the activation of the AMP-activated protein kinase, MAPK, and peroxisome proliferator-activated receptor pathways. On the other hand, contradictory results exist concerning the metabolic and antiproliferative effects of adiponectin in human breast cancer cells that express estrogen receptors [154].

A chronic low-grade inflammatory condition is often associated with obesity-related IR and type 2 diabetes, and may provide an additional link between these metabolic disorders and cancer, in which cytokines and other inflammatory mediators may initiate and sustain cancer progression. For instance, TNF-α has been found in both acute and chronic inflammatory states, where it seems to promote and amplify IR by inhibiting INSR signaling [170,171]. In addition, higher levels of circulating TNF-α have been associated with tumorigenesis in overweight IR patients, in which its anti-apoptotic effect was most likely due to the activation of the NFκB pathway [172].

Interleukin 6 (IL-6) is an inflammatory cytokine involved in both metabolic and neoplastic disorders. Correlation between adipose tissue expression and serum concentration of IL-6 obesity-related IR and type 2 diabetes has been reported [173,174]. It has, also, been demonstrated that IL-6 may induce cancer proliferation via STAT signaling, while blocking, at the same time, the host anti-tumor immune response [175]. Higher levels of IL-6 have been found in overweight and obese women with IR and early-stage breast cancer [176]. Moreover, compared to men with benign conditions, levels of circulating IL-6 have been found to be elevated in patients with prostate cancer [177]. However, it is possible that the association of IL-6 with prostate cancer risk could be modified by BMI, as increasing IL-6 was positively associated with prostate cancer in normal-weight subjects, but inversely associated with prostate cancer in overweight/obese men [177]. It must be said that these results were unadjusted for the various degrees of IR and, therefore, this may have confounded these associations [177].

MCP-1 is a chemoattractant for macrophages and other immune cells, capable of driving their migration to the adipose tissue. MCP-1 overexpression in fat leads to the development of inflammation and can participate in malignant transformation and/or cancer progression [178,179,180]. During obesity, macrophages infiltrate the adipose tissue and release TNF-α, which in turn triggers the NFκB and JNK-MAP4K4-AP1 (activator protein 1) pathways, thus amplifying the mitogenic signals [181,182,183].

Deregulated lipolysis and overproduction of free fatty acids (FFA) are some of the main mechanisms implicated in the disruption of endocrine function of adipocytes. Hypertriglyceridaemia and associated high circulating levels of FFA are a hallmark of adipose tissue expansion, resulting in lipotoxicity and development of IR [184]. In muscle, excess FFA may lead to the activation of PKC isoforms theta, beta2, and delta, with subsequent reduction of INSR and IRS-1 activity. In addition, high FFA levels can amplify the pro-inflammatory response via NFκB signaling and increased secretion of pro-inflammatory cytokines such as IL-6, IL-1, TNF-α, and MCP-1 [185]. Furthermore, NFκB may induce the expression of target genes involved in cell migration, anti-apoptosis, angiogenesis, and cell proliferation. Finally, it should be mentioned that IR and hyperglycemia may boost the formation of advanced glycation end (AGE) products [186], which are important triggers of oxidative stress and ROS-mediated DNA damage, in addition to the possibility that AGE precursors may also exert cellular damage by altering endogenous protein structure and function [187].

## 5. Epigenetic Modifications

The term “epigenetics” indicates the heritable changes in gene function that do not rely on changes in the DNA sequence [188]. Epigenetic changes can be triggered by a variety of environmental factors or “stressors”, including diet, physical activity, stress, smoking, and toxins [189], and as such they can be involved in a variety of physiological and pathological biological processes. Therefore, epigenetics is emerging as an important mechanistic link between environmental factors and altered gene activity, especially in chronic diseases such as obesity, type 2 diabetes, and cancer, in the pathogenesis of which aberrant gene–environment interactions have been implicated [190]. Many recent studies, in this context, have found that epigenetic modifications are likely involved in oncogenesis, either indirectly by promoting obesity, IR, and type 2 diabetes, or by means of common pathogenetic traits between these metabolic disorders and cancer. Epigenetic modifications are reversible and modifiable and, thus, they represent an excellent potential target for therapeutic intervention by either non-pharmacological (i.e., lifestyle changes) and/or pharmacological measures [191]. Important epigenetic mechanisms involve DNA methylation, posttranslational histone modification (PTM), and regulation of gene expression by non-coding RNA (ncRNAs) [188].

In mammals, DNA methylation consists in the addition of methyl groups to the DNA, playing a key role in the regulation of transcription as, in general, hypermethylation of promoters turns off gene expression, whereas hypomethylation would favor gene expression [192,193]. It has been observed that the methylation of genes related to endoplasmic reticulum stress was inversely associated with the HOMA-IR index [194], a surrogate marker of IR. In addition, the catalytic activity of DNA methyltransferase 3a has been found to be involved in the induction of IR, both in vitro and in vivo, in adipose tissue [195]. Among the numerous differential DNA methylation patterns associated with obesity in epigenome-wide association studies [193,196], the up-regulation of SOCS3 following hypomethylation has been recently reported to increase the risk of IR and diabetes [197]. Moreover, differentially methylated CpG sites during the first year of life have been found to be associated with early weight gain and later childhood obesity, thus suggesting that early-life epigenetic changes may predict an individual’s future risk of obesity and obesity-related comorbidities [198]. However, the significance of DNA methylation in the development of obesity and the risk to develop type 2 diabetes has not always been shown [193], and there is some suggestion that variation in DNA methylation can occur in response to changes in BMI [198].

Methylation, acetylation, and deacetylation, by far the most widely researched PTMs of histones, largely influence chromatin packaging and, ultimately, gene transcription [199]. Histone PTMs are involved in the induction and progression of various chronic disorders, including obesity, type 2 diabetes, and cancer [193,200]. In recent years, some specific histone marks and their PTMs have been identified [201,202], which may represent novel therapeutic targets for these diseases [193]. For example, monomethylation of lysine 4 of histone 3 on the NF-kB p65 promoter, which depends on the activity of methyltransferase Set-7, is one of the epigenetic signatures of peripheral blood mononuclear cells from patients with type 2 diabetes and relates with proinflammatory phenotypic changes and vascular dysfunction. In vitro silencing of Set-7, and thus prevention of its methyltransferase activity on histones, abolishes the inflammatory and prooxidant signaling of NF-kB [201].

Nonetheless, some members of the non-coding genome, comprising micro RNAs (miRNAs) and long ncRNAs (lncRNAs) of 18–25 and over 200 nucleotides in length, respectively, represent the most relevant mechanism of post-transcriptional regulation, and their biology has been put at the center of current basic research. Via binding to 3′-untranslated regions (3′-UTR) of target mRNAs and influencing the decay of transcripts, miRNAs typically act as repressors of gene expression [203], whereas a variety of mechanisms of action have been described for lncRNAs. These include: (i) direct interaction with DNA, coding RNA molecules and chromatin proteins (i.e., histones); (ii) binding of small RNAs or RNA-binding proteins; (iii) competition with endogenous RNA for binding to miRNAs; (iv) trafficking of RNA-binding proteins to the effector molecules [204,205]. Recently, a large number of studies have highlighted the roles of miRNAs and lncRNAs in human diseases, including obesity, IR, and diabetes, cardiovascular disease, inflammation, and cancer [204,205,206], so that various miRNAs and lncRNAs have been identified as biomarkers for these diseases. Herein, we discuss some of them.

**Figure 3:**
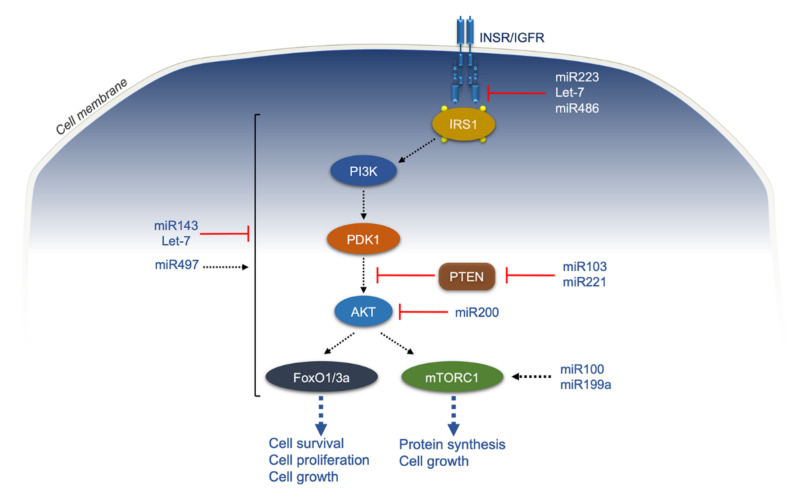
Actions of miRNAs in cancer and insulin resistance. INSR/IGFR, insulin receptor/insulin-like growth factor receptor; IRS1, INSR substrate 1; PI3K, phosphoinositide 3-Kinase; PDK1, phosphoinositide-dependent protein kinase-1; PTEN, phosphatase and TENsin homolog deleted on chromosome 10; AKT, Ak strain transforming/protein kinase B; FoxO1/3a, forkhead box protein O1/3a; mTORC1, mammalian target of rapamycin complex 1; black dotted arrows, activation; red lines, inhibition; blue dotted arrow, biological effects of the downstream signaling.

The paralogous miRNAs, miR-103, and miR-107, which differ only at one nucleotide residue close to their 3′ ends, are not only upregulated in obesity-related IR and impaired glucose homeostasis [207], but are also of central importance in the pathogenesis of several malignancies, including breast, liver, and colorectal cancer, because of their inhibitory effects on RNA-processing enzyme Dicer and the tumor suppressor Axin2 [208,209]. In addition, miR-103 can promote colorectal cancer by targeting the tumor suppressor phosphatase and tensin homolog deleted on chromosome 10 (PTEN) [210], one of the most frequently mutated or deleted tumor suppressor genes in IR [211], obesity, and type 2 diabetes [212], as well as in a variety of human tumors [213] (Figure 3).

The highly homologous miR-221 and miR-222, significantly overexpressed in several types of cancers, are encoded from a gene cluster located on chromosome Xp11.3 and transcribed as a single lncRNA precursor [214]. High expression levels of miR-221 were observed in adipose tissue from obese individuals [215], in which it upregulated the levels of several proteins involved in fat metabolism, and downregulated adiponectin receptor and signaling, thereby impairing insulin sensitivity and predisposing to the development of type 2 diabetes. In addition, miR221 downregulates the transcription factor v-ets erythroblastosis virus E26 oncogene homolog 1 (ETS1), involved in both the regulation of cytokine and chemokine production and VEGF-induced angiogenesis [215]. As in the adipose tissue of patients with obesity, an inverse correlation between ETS1 and miR-221 expression levels has been reported in many malignancies, thus indicating that miR-221 may induce oncogenesis via ETS1 [215]. It has also been observed that in triple-negative breast cancers, the highly expressed miR-221 and miR-222 directly target and suppress Dicer [216]. Evidence also exists that this oncogenic miRNA cluster modulates the PTEN/Akt transduction pathway and downregulates the tumor suppressor ARH1, whose deficiency may promote pancreatic and prostate cancer [217,218], and can be found in more undifferentiated neuroblastoma cell lines [219] (Figure 3). A pathogenic role of miR-221 and miR-222 has been described in other types of cancer, including glioma, thyroid, breast, and gastrointestinal tumors [220], whereas miR-221 and miR-222 overexpression has been associated with increased tumor grade and poorer prognosis [221].

MiR-29 family members (miR-29a, miR-29b and miR-29c) are implicated in numerous pathophysiological processes, including IR, diabetes, and cardiovascular disease [222]. In particular, in silico studies have predicted miR-29a as a key regulatory hub in gene networks implicated in beta cell function and the pathogenesis of type 2 diabetes [223]. Consistently, miR-29 paralogs were found to be overexpressed in the skeletal muscle, fat, and liver of diabetic rats [224]. It has been suggested that miR-29 family members may promote IR and type 2 diabetes through the downregulation of proteins involved in insulin signaling, such as CAV1 and insulin-induced gene 1 (Insig1) [225], and by targeting syntaxin-1, a protein that plays a positive role in both GLUT4 function and insulin exocytosis [224]. In cancer, miR-29 family members can either act as oncogenic factors or tumor suppressors, depending on the cell of origin and tissue specificity [226]. For example, miR-29 members are up-regulated in breast cancer, pleural mesothelioma, colorectal cancer, and diffuse large B lymphoma [226], where they correlate with an advanced neoplastic stage and act as tumor activators [227]. In particular, it has been demonstrated that miR-29a is upregulated in ER- breast cancer, as compared with ER+ breast cancer tissues and adjacent non-tumor tissues, and this correlates with tumor metastasis and overall short survival [228]. From a mechanistic perspective, miR-29a favors EMT and breast cancer cell growth and migration by targeting the tumor suppressor Ten Eleven Translocation 1 (TET1) [228], which plays a role in response to environmental and endogenous factors, including nutrition, lifestyle, chemicals, and air pollutants exposure. On the contrary, low miR-29 levels have been found in gastric and esophageal carcinomas [226].

Let-7 and its family members, including let-7a, b, c, d, e, f, g, i, miR98, and miR-202, have been found to play important roles in tumor suppression by blocking the expression of several oncogenes. Let-7 miRNAs recognize and bind the same region (except miR-202), modulating the degradation and stability of their mRNA targets [229]. On the other hand, the RNA-binding proteins Lin28a/b promote tumor growth and progression by inhibiting let-7 biogenesis. However, let-7 has also been found to be implicated in the regulation of metabolic processes, and a positive correlation between let-7a and b levels has been observed in skeletal muscle of patients with type 2 diabetes [230]. Experiments carried out in transgenic mice showed that both Lin28a and Lin 28b may play an important role in peripheral insulin sensitivity, and tissue-specific loss of Lin28a along with the over-expression of let-7 caused impaired glucose tolerance and IR [230]. Research on stem cells from adipose tissue have shown that sustained expression of Lin28 restored glucose metabolism, counteracting let-7 activity [231]. At the molecular level, let-7 performs its main regulatory function via repression of factors regulating insulin-PI3K-mTOR signal transduction and inhibiting IGF1R, INSR, and IRS2 expression [232] (Figure 3). Further studies, in this context, have demonstrated that IGF1R and INSR expression was restored following forced downregulation of let-7 miRNA [233], while a recent report of our group identified miR-128 as a hypoxia-induced miRNA, whose increase in obesity negatively affects INSR [20]. In addition, microarray assays have revealed an inverse correlation between let-7a, c, g, and TNF-α levels in patients with type 2 diabetes compared to control subjects [234].

The miRNA miR-223 has been implicated in IR and obesity by modulating STAT signaling pathways, the toll-like receptor 4 (TLR4), and the F-box/WD repeat-containing protein 7 (FBXW7), a negative regulator of adipogenesis and a tumor suppressor in many cancer types [235]. IGF1R and PI3K/Akt are direct targets of miR-223 [236]. Forced down-regulation of miR-223 leads to PI3K/Akt pathway activation and cancer cell growth [237]. In addition, miR-223 has been one of the first miRNAs found to be secreted, thus reinforcing the hypothesis that miRNAs can operate as circulating mediators of systemic insulin sensitivity and glucose homeostasis.

Under aerobic conditions, the increased conversion of glucose into lactate (via glycolysis) allows cancer cells to produce several metabolic products such as Acetyl-CoA, glycolytic intermediates, and ribose for the biosynthesis of fatty acids, amino acids, and nucleotides [238,239]. In this regard, a large number of miRNAs were found to be involved in the regulation of PI3K/Akt/mTOR activity, a pathway promoting cell growth, and protein and lipid biosynthesis in cancer cells [240,241]. In the liver of obese mice, the upregulation of miRNA 143 caused a strong suppression of oxysterol-binding-protein-related protein 8, which in turn blocked the capability of insulin to stimulate Akt [242,243]. On the other hand, an inverse correlation was established between the expression of miRNA 200 family members and Akt activity, resulting in higher EMT and greater cancer stem-cell-like properties [244]. In addition, in ovarian cancer cells, a significant relationship was found between miRNA 100 and mTOR signaling [245], whereas an involvement of miRNA 199a-3p was reported in several human malignancies, in which downregulation of this miRNA paralleled the amplification of mTOR’s effects on tumor cell survival [246] (Figure 3).

MicroRNA miR-486-5p was downregulated under conditions of prediabetes [247], and its expression was also altered in various kind of cancers [248]. It has been shown that miR-486 may act either as a tumor-suppressor or an oncogene, depending on the cell context [249,250,251,252,253]. IGF1, IGF1R, and p85α were considered to be targets of miR-486 and, in fact, miR-486 overexpression inhibited Akt signaling and FoxO activity, thereby amplifying its anti-apoptotic effect in both in vitro and in vivo conditions [249] (Figure 3).

Finally, miRNA-497, which represses mitochondrial uncoupling protein 2, regulates pancreatic insulin secretion in response to postprandial hyperglycemia [254]. MiRNA-497 has been found to be downregulated in several types of cancers, including breast, gastric, pancreas, and colorectal cancer. In particular, in colorectal cancer, reduced expression of miRNA-497 has been associated with overexpression of IGF1R and IRS1 with consequent activation of PI3K/Akt signaling, thus contributing to tumor progression, growth, and survival [255] (Figure 3). Similar effects were also observed in non-small-cell lung cancer cells and in cervical cancer tissues [256,257], whereas upregulation of miRNA-497 was detected in human glioma cells, suggesting a dual role of this miRNA as tumor suppressor or oncomiR, depending on tissue type or context [258].

Concerning lncRNAs, upregulation of HOTAIR (Hox transcript antisense intergenic RNA) [259], MEG3 (maternally-expressed gene 3) [260], or Gomafu, also known as MIAT (myocardial-infarction-associated transcript) [261] have been shown to cause IR by increasing the expression of FoxO1, and promoting hepatic gluconeogenesis. Additionally, the circulating levels of lncRNA growth-arrest-specific 5 (GAS5) have been found to be reduced both in diabetic patients as well as in a type 2 diabetic mouse model, suggesting an involvement of this lncRNA in the pathogenesis of this disease [262,263]. Other studies showed that lncRNA GAS5 can regulate cell proliferation, apoptosis, and invasion, and its expression is reduced in various cancer types, including lung and breast cancer [264,265]. Intragenic noncoding RNA (IRAIN) is a lncRNA that acts as a putative tumor suppressor affecting IGF1R expression [266,267]. IRAIN expression was downregulated in breast cancer. Notably, when its levels were restored via genetic engineering methods, a significant reduction of cancer migration and proliferation was reported [268]. Metastasis-associated lung adenocarcinoma transcript 1 (MALAT1) was the first lncRNA with a designated role in lung adenocarcinoma [269]. It has been reported that lncRNA MALT1 plays a role in IR and the pathophysiology of type 2 diabetes by interacting with the transcription factor Nrf2, thus promoting ROS accumulation, which, in turn, alters IRS and PI3K/Akt signaling pathways. In addition, it has been documented that dysregulation of MALAT1 might contribute to the development of diabetic retinopathy, a leading cause of vision loss and blindness [270]. The antisense noncoding RNA in the INK4 locus (ANRIL) is a lncRNA that plays a role in the epigenetic silencing of genes. Its locus at chromosome 9p21 has been involved in several human diseases, including atherosclerosis and coronary artery disease, as well as cancer and type 2 diabetes [271,272].

## 6. Gut Microbiota and Cancer

The microbiota represents a multitude of microorganisms inhabiting the human gut, which establish a durable and specific relationship with the host immune system and mucosal epithelial cells [273]. Recent lines of evidence indicate that gut microbiota exerts fundamental effects on host health and well-being through a series of mechanisms that can be related to the following processes: (1) regulation of immune cell function; (2) prevention of growth, virulence, and colonization of pathogens; (3) transformation or elimination of toxic substances; (4) production of fermentation-derived metabolites neutralizing the intestinal pH; (5) enhancement of fecal bulk and dilution of toxic substances; (6) absorption of beneficial dietary elements and minerals [274]. It has been ascertained that nutritional changes, aging, medications, stress, and lifestyle can profoundly affect the microbiota, leading to inflammatory and pathophysiological states of the intestine and distant organs and tissues. Aberrant gut microbiota composition and function (dysbiosis) has been associated with obesity, IR, and both type 1 and type 2 diabetes with probable causal significance [275,276,277]. It is well recognized that microbiota can impact on gut and systemic host metabolism through several mechanisms such as the maintenance of gut barrier integrity, the ability to absorb energy from digested nutrients, the production of metabolites (i.e., short chain fatty acids (SCFAs), secondary bile acids, endotoxins) and gut-derived hormones (i.e., GLP-1, GIP, PPY) that affect satiety and/or peripheral insulin sensitivity, and the modulation of epigenetic modifications [278]. Therefore, it may come as no surprise that gut dysbiosis could be linked to various human diseases, including cancer, by influencing the genomic stability of host cells through the modulation of many different signaling pathways [279]. Previous studies have reported a correlation between low gut microbiota richness (i.e., low bacterial gene count) and metabolic dysfunction, including IR, dyslipidemia, excess adiposity, and inflammation [275]. In addition, reduced circulating levels of the gut-microbiota-derived metabolite trimethylamine N-oxide (an early biomarker of adipose dysfunction), as induced by low-calorie diet, was associated with enhanced insulin sensitivity and improved glucose tolerance in obese adults that underwent caloric restriction [280]. A. muciniphila, a mucin-degrading bacterium residing in the intestinal mucus layer, has been proven to be less abundant in HFD-fed obese mice with IR and type 2 diabetes, in which, however, augmentation of A. muciniphila through prebiotic feeding was able to revert HFD-induced metabolic disorders [281]. SCFAs produced in the gut following microbial fermentation of dietary fibers, not only provide ~10% of a host’s daily energy needs [282], but also affect host metabolism. Accordingly, it has been shown that fecal levels of the SCFAs propionate and acetate, as well as of total SCFAs, are inversely correlated with insulin levels and HOMA-IR in patients with type 2 diabetes [283]. Furthermore, it has been observed that the gut-microbiota-derived SCFA butyrate inhibits histone deacetylase 3 (HDAC3), and may have regulatory effects on host metabolic control and cytokine release via the negative modulation of histone acetylation status in intestinal epithelial cells. Compared to wild-type mice, Hdac3-knockout mice on HFD feeding gained less weight, and had less liver fat and smaller adipocytes, being resistant to HFD-induced obesity [284]. Consistently, on HFD mice, supplementation of SCFA butyrate ameliorated weight gain and IR [285].

Recent studies have shown that diet-induced obesity due to increased intake of saturated fats and low fiber content was associated with changes in Firmicutes and Bacteroidetes species composition, two dominant bacterial phyla in the large intestine microbiota, which are involved in the breakdown of dietary fibers and polyphenols, with consequent alterations in colonic fermentation patterns and SCFAs components [286]. It has been reported that obesity-related imbalance of microbiota may promote the growth of detrimental bacterial species and gut dysbiosis [287], which, in turn, impacts on estrogen metabolism. Indeed, most bacteria in the gut exhibit β-glucuronidase enzyme activity, which can hydrolize conjugated estrogens into active free estrogens, thereby allowing their reabsorption and enterohepatic circulation [288]. When this process is impaired as a result of gut dysbiosis, the decrease in β-glucuronidase enzyme activity results in reduced levels of circulating estrogens, whereas the opposite occurs when this enzyme activity is increased. This observation supports well the assumption that a close connection might indeed exist between gut microbiota, dysmetabolism, and the higher predisposition to some estrogen-driven malignancies, such as breast cancer and postmenopausal endometrial cancer [289]. Gaining insights into the relationship between diet, gut microbiota, and host on cancer risk might offer a potential for effective prevention and identification of more efficient anticancer strategies.

## 7. Antidiabetic Medications as Potential Anticancer Agents

Alongside nutrition therapies, pharmacological interventions for diabetes have been suggested to influence cancer risk in affected patients. However, if the use of insulin analogues and conventional insulin secretagogues, which lead to increased levels of circulating insulin [290], has been associated with increased risk of cancer [121,122,291,292], metformin and thiazolidinediones (TZDs) may have the potential to prevent tumor development.

### 7.1. Metformin

Metformin is widely regarded as the first-line drug for most patients with type 2 diabetes [293]. Furthermore, in view of its safe profile and low cost, metformin is also used to ameliorate IR even in nondiabetic patients, such as PCOS patients. Apart from its glucose-lowering effects and the clinically proven benefits on both pregnancy rates and hyperandrogenic traits of PCOS, there is increasing epidemiologic evidence that patients using metformin have reduced risk of developing cancer, thus highlighting the preventive and therapeutic potential of this drug against malignancies [294,295]. In this regard, post-menopausal diabetic women under metformin treatment had lower risk of invasive breast cancer compared to post-menopausal diabetic women on a different oral hypoglycemic drug [296,297]. Importantly, these benefits were independent of diabetes and, to some extent, of breast tumor subtype, suggesting the use of metformin as a chemopreventive agent in breast cancer, either alone or in combination with chemotherapeutic drugs and/or radiation therapy [298]. Besides breast cancer, the beneficial effects of metformin on the urothelial carcinoma of the bladder, one of the deadliest neoplastic diseases worldwide [299], have also been documented. Preclinical in vivo studies, using syngeneic orthotopic murine models of bladder cancer, have demonstrated a significant inhibitory effect of metformin on tumor growth following intravesical drug instillation, without obvious side effects such as vomiting, gastrointestinal disturbances, or hematuria [300]. Findings in this context suggest that even oral metformin may be a promising and practical alternative to the current adjuvant intravesical treatment in patients with non-muscle invasive bladder cancer [301]. At the same time, observational studies indicate that the use of oral metformin is associated with improved recurrence-free survival and bladder-cancer-specific survival in patients with diabetes following radical cystectomy [302]. On the other hand, despite a wealth of preclinical and observational data linking metformin to improved prostate-cancer-related outcomes [303,304,305], the addition of metformin to abiraterone showed no significant clinical benefit on survival in patients with metastatic castration-resistant prostate cancer, raising a controversy about the use of metformin in this setting [306]. In the last years, several meta-analyses have investigated the effect of metformin on the incidence and prognosis of gynecological tumors, such as ovarian, endometrial, and cervical cancer [307,308,309]. Although conflicting results exist in the literature with regard to metformin and endometrial cancer, and heterogeneity across studies is substantial, it has been estimated that there is a significant lower incidence and better prognosis of gynecological cancers in patients with metformin therapy. Moreover, several recent meta-analyses indicate that metformin may also reduce the incidence of lung cancer [310,311] and increase lung cancer survival in metformin-treated type 2 diabetic patients [311,312]. Finally, limited data from inconclusive meta-analyses suggest that metformin might improve all-cause mortality in certain cancer types [313], including gastric [314], pancreatic [315], and colorectal cancer [316], and reduce breast cancer-specific mortality [313].

**Figure 4:**
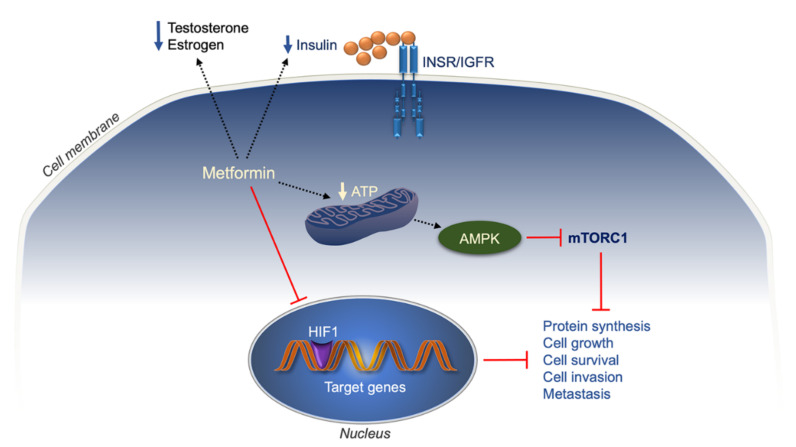
Anticancer action of metformin. INSR/IGFR, insulin receptor/insulin-like growth factor receptor; AMPK, 5′ adenosine monophosphate-activated protein kinase; mTORC1, mammalian target of rapamycin complex 1; HIF1, hypoxia-inducible factor 1; black dotted arrows, activation; red lines, inhibition.

However, in spite of wide research in this field, the mechanism(s) underlying the anticancer effects of metformin are yet largely unknown. Apart from metformin-induced inhibition of mitochondrial electron transport chain (ETC) and ATP synthesis, it has been proposed that metformin may regulate the AMPK/mTORC1 pathway by multiple, mutually nonexclusive mechanisms that might prevent tumor growth, and are not necessarily dependent on the inhibition of ETC and intracellular ATP levels [317] (Figure 4). Furthermore, metformin inhibits the expression of HIF-1α in several human cell lines, including the multidrug-resistant cell line Bel-7402/5-FU of hepatocellular carcinoma [318] and the prostate cancer cell line PC3 [319]. HIF-1α transactivates genes whose protein products are involved in many aspects of cancer biology, including cell immortalization and proliferation, maintenance of stem cells, genetic instability, energy metabolism, angiogenesis, EMT, cancer cell invasion and metastasis, and chemotherapy resistance [320,321]. Thus, based on these studies, it is evident that agents blocking HIF-1α expression or inhibiting HIF-1α activity may be useful in improving current cancer therapies [321]. Other proposed mechanisms that may account for the anticancer action of metformin appear to be indirect, probably dependent on the metformin-induced reduction of plasma glucose and insulin levels and its beneficial effects on some IR mitogenic biomarkers, such as IGF1 and IGFBP3 [322], leading to decreased activation of INSR (and INSR/IGF1R hybrid receptors) and attenuation of growth and proliferation of a subset of tumors for which chronic hyperinsulinemia may offer a growth advantage. Another beneficial mechanism that metformin may have in breast cancer relies on its ability to reduce the bioavailability of sex hormones, androgens, and estrogens. Actually, high circulating levels of testosterone and estrogen are associated with increased risk of breast cancer [323,324]. Therefore, the reduction of sex hormones by metformin treatment might have clinical relevance.

### 7.2. Thiazolidinediones

**Figure 5:**
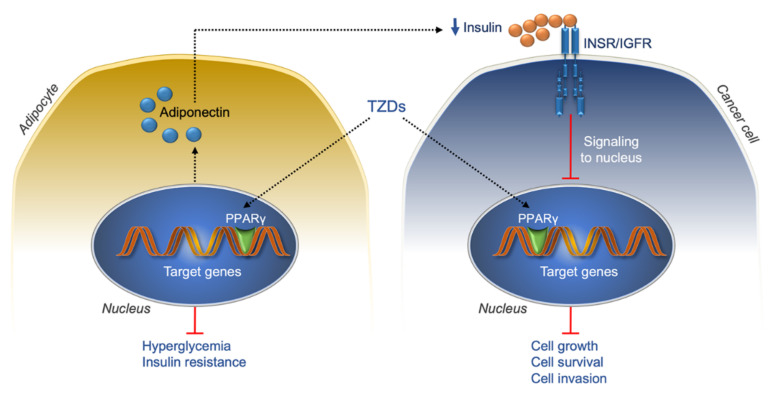
Anticancer actions of TZDs. INSR/IGFR, insulin receptor/insulin-like growth factor receptor; TZD, thiazolidinediones; PPARγ, proliferator-activated receptor γ; black dotted arrows, activation; red lines, inhibition.

Thiazolidinediones (TZDs) are a class of insulin-sensitizing drugs used alongside diet and exercise for treatment of type 2 diabetes. However, because of side effects and safety concerns, the use of TZD has been limited and most often used when other insulin sensitizers (i.e., metformin) don’t work [325,326]. In spite of the beneficial effects of TZDs on IR and glucose homeostasis (at least in part, a consequence of TZD-induced PPARγ receptor activation, adipocyte fatty acid uptake, and adiponectin secretion) [327], certain epidemiological data, but not all [328], have also shown that long-term treatment with the TZD pioglitazone, is associated with an increased risk of bladder cancer in patients with type 2 diabetes [329,330]. Unlike pioglitazone, rosiglitazone, another TZD PPARγ agonist, did not show any significant risk of bladder cancer, supporting the notion that cancer risk of TZDs is compound-specific and not class-specific [327]. On the other hand, antiproliferative effects of TZDs on breast, prostate, and other types of cancer, have been evidenced in malignant human cell lines and rodents [327,331,332], together with the results that combination with PPARγ agonists may improve some cancer therapies [327]. These observations are in line with those from a large meta-analysis of clinical trials, showing not only that there was no relationship between TZDs and increased risk of overall malignancies, but also that TZD use may confer protection against cancer (pooled OR = 0.85 (95% CI: 0.73–0.98)) [326]. Nonetheless, the results of subgroup analyses, stratified by TZD compounds and tumor site, did not exclude an increased risk of bladder cancer after pioglitazone use, while the risk of breast cancer was decreased [326]. At the same time, the use of rosiglitazone was associated with a modest but significantly reduced risk for colorectal cancer, but not breast cancer [326]. An updated meta-analysis of clinical studies, either in the form of randomized controlled trials or case-control and cohort studies, did not show any significant relationship between use of TZDs and risk of incident breast cancer among women with type 2 diabetes [333]. However, among the included studies, the total number of incident breast cancer cases was small, while the reference control group was rather heterogeneous, as it consisted of patients with a variety of antidiabetic medication treatments with potential chemopreventive properties, such as metformin [333]. Besides the PPARγ-dependent insulin-sensitizing effects, which reduce circulating levels of insulin and free IGF1, and downregulate the insulin/IGF1 signaling pathway that plays a role in cancer initiation/progression, other mechanisms, independently of PPARγ activation, could contribute to the antitumor activity of TZDs. Although current understanding in this area is limited, and the results from basic research often look conflicting, PPARγ-independent mechanisms have been also reported [334,335] (Figure 5).

## 8. Conclusions

In the last years, many basic and clinical studies have provided extensive evidence that supports the idea of a strong association between obesity and diabetes and a greater risk of developing cancer. A wide variety of molecular mechanisms and signaling pathways to explain this association have been proposed in this review article, in which IR, a hallmark of type 2 diabetes and a characteristic feature of obesity and obesity-related diseases, may represent a mechanistic pathway implicated in cancer pathophysiology and, as such, an intervention target in affected cancer patients. Chronic sustained hyperinsulinemia, INSRs, IGF1Rs, and INSR/IGF1R hybrids, in addition to chronic inflammation, ncRNAs, and microbiota have been proposed as factors that may play a role in all tumor stages. As reviewed here, there is no one single factor that explains the link between IR and cancer, and further investigations and clinical association studies are needed for both the identification of new underlying molecular and cellular mechanisms of interaction between IR and cancer, and a better understanding of what is already known about this topic. It is probable that in the future, efforts will be targeted on the development of novel diagnostic and therapeutic strategies. However, at the same time, it becomes crucial to recognize those patients who might rather benefit from more effective personalized therapies with molecularly targeted drugs.

## Figures and Tables

**Table 1:** Selected meta-analyses exploring the association between diabetes mellitus and cancer risk.

Cancer Site	Type 1 Diabetes	Type 2 Diabetes	
	Incidence	Incidence	Mortality
All sites	▲ RR = 1.29 (1.09–1.52) [83]	▲ RR = 1.10 (1.04–1.17) [84]	▲ RR = 1.16 (1.03–1.30) [84]
Biliary tract		▲ RR = 1.43 (1.18–1.72) [85]	
Bladder	⬄ RR = 0.98 (0.88–1.09) [83]	▲ RR = 1.35 (1.17–1.56) [86]	
Breast	⬄ RR = 0.91 (0.86–0.95) [83]	▲ RR = 1.20 (1.12–1.28) [87]	▲ HR = 1.51 (1.34–1.70) [88] HR = 1.37 (1.34–1.41) [89]
Cervix	⬄ RR = 1.24 (0.99–1.56) [83]		▲▲ HR = 1.59 (1.35–1.87) [90]
Colon-rectum	⬄ RR = 0.90 (0.61–1.31) [83]	▲ RR = 1.27 (1.21–1.34) [91]	▲ HR = 1.18 (1.12–1.24) [92]
Endometrium	▲▲ RR = 1.67 (1.22–2.30) [83] ▲ RR = 1.42 (1.27–1.58) [95]	▲▲ RR = 1.63 (1.41–1.88) [93]	⬄ RR = 1.23 (0.78–1.93) [94]
Esophagus	▲ RR = 1.06 (1.02–2.42) [83]	▲ RR = 1.30 (1.12–1.50) [94]	
Hematological malignancies	⬄ RR = 1.01 (0.88–1.16) [83]	▲ OR 1.22 (1.03–1.44) [96]	
Kidney	▲ RR = 1.37 (1.23–1.52) [83]	▲ RR = 1.40 (1.16–1.69) [97]	⬄ RR = 1.12 (0.99–1.20) [97]
Liver	▲▲▲ RR = 2.35 (2.12–2.61) [83]	▲▲ RR = 1.60 (1.38–1.87)] [98] ▲ RR = 1.49 (1.32–1.70) [85,99]	
Lung	▲ RR = 1.09 (1.02–1.17) [83]	▲ HR = 1.14 (1.09–1.20) [100]	▲ HR = 1.33 (0.87–2.03) [101]
Ovary	▲ RR = 1.17 (1.04–1.32) [83]	▲ RR = 1.17 (1.02–1.33) [102,103]	
Pancreas	▲ RR = 1.34 (1.18–1.52) [83]	▲▲ RR = 1.94 (1.66–2.27) [104]	▲ RR = 1.67 (1.30–2.14) [93]
Prostate	⬄ RR = 1.15 (0.30–4.41) [83] ▼ RR = 0.56 (0.51–0.61) [95]	▼ RR = 0.83 (0.79–0.88) [93]	▲ HR = 1.50 (1.25–1.79) [105]
Stomach	▲ RR = 1.44 (1.29–1.61) [83]	▲ RR = 1.19 (1.07–1.32) [106] ⬄ RR = 1.9 (0.98–1.22) [107]	▲ RR = RR 1.29 (1.04–1.59) [106]
Thyroid	▲ RR = 1.40 (1.19–1.66) [83]	▲ RR = 1.17 (0.99–1.39) [108]	

RR, risk ratio; OR, odds ratio; HR, hazard ratio; ▲, RR 1.1–1.49; ▲▲, RR 1.5–1.99; ▲▲▲, RR > 2.0; ▼, RR < 1.0; ⬄, NS.
